# Evaluation of the Endorsement of the Preferred Reporting Items for Systematic Reviews and Meta-Analysis (PRISMA) Statement on the Quality of Published Systematic Review and Meta-Analyses

**DOI:** 10.1371/journal.pone.0083138

**Published:** 2013-12-26

**Authors:** Nikola Panic, Emanuele Leoncini, Giulio de Belvis, Walter Ricciardi, Stefania Boccia

**Affiliations:** 1 Section of Hygiene, Institute of Public Health, Università Cattolica del Sacro Cuore, Rome, Italy; 2 Faculty of Medicine, University of Belgrade, Belgrade, Serbia; 3 University Clinical-Hospital Center “Dr Dragisa Misovic-Dedinje”, Belgrade, Serbia; 4 IRCCS San Raffaele Pisana, Rome, Italy; Brunel University, United Kingdom

## Abstract

**Introduction:**

PRISMA statement was published in 2009 in order to set standards in the reporting of systematic reviews and meta-analyses. Our aim was to evaluate the impact of PRISMA endorsement on the quality of reporting and methodological quality of systematic reviews and meta-analyses, published in journals in the field of gastroenterology and hepatology (GH).

**Methods:**

Quality of reporting and methodological quality were evaluated by assessing the adherence of papers to PRISMA checklist and AMSTAR quality scale. After identifying the GH journals which endorsed PRISMA in instructions for authors (IA), we appraised: 15 papers published in 2012 explicitly mentioning PRISMA in the full text (Group A); 15 papers from the same journals published in 2012 not explicitly mentioning PRISMA in the full text (Group B); 30 papers published the year preceding PRISMA endorsement from the same journals as above (Group C); 30 papers published in 2012 on the 10 highest impact factor journals in GH which not endorsed PRISMA (Group D).

**Results:**

PRISMA statement was referred in the IA in 9 out of 70 GH journals (12.9%). We found significant increase in overall adherence to PRISMA checklist (Group A, 90.1%; Group C, 83.1%; p = 0.003) and compliance to AMSTAR scale (Group A, 85.0%; Group C, 74.6%; p = 0.002), following the PRISMA endorsement from the nine GH journals. Explicit referencing of PRISMA in manuscript was not associated with increase in quality of reporting and methodological quality (Group A vs. B, p = 0.651, p = 0.900, respectively). Adherence to PRISMA checklist, and the compliance with AMSTAR were significantly higher in journals endorsing PRISMA compared to those not (Groups A+B vs. D; p = 0.003 and p = 0.016, respectively).

**Conclusion:**

The endorsement of PRISMA resulted in increase of both quality of reporting and methodological quality. It is advised that an increasing number of medical journals include PRISMA in the instructions for authors.

## Introduction

The number of systematic reviews and meta-analyses published in scientific journals is increasing every year [1]. Nowadays these tools represent a basis to develop recommendations and guidelines, and a valuable source in assisting physicians in the decisions making process [2]–[3]. In view of the high level of evidence they are expected to offer, the methodological quality of the systematic reviews and meta-analyses represents a critical issue.

In the past some studies reported a low quality of the systematic reviews and meta-analyses published in some of the leading medical journals [4], [5]. That led to development of the QUality Of Reporting Of Meta-analysis (QUOROM) statement in 1996 [6], which was expected to set some standards in drafting of meta-analysis of randomized clinical trials [7]. Later in 2009, the Preferred Reporting Items for Systematic reviews and Meta-Analysis (PRISMA) statement revised the QUORUM guidelines in order to address several conceptual and practical advances in the reporting of systematic reviews and meta-analyses of observational and experimental studies [8]. Currently, around 30% of medical journals publishing systematic reviews have endorsed PRISMA statement in their author's guidelines [9].

After the release of PRISMA, however, no attempts have been done to evaluate the impact of PRISMA endorsement on the quality of reporting and the methodological quality of the published systematic reviews and meta-analyses. Even though PRISMA was not intentionally developed to improve the conduction of systematic reviews and meta-analyses [7], it is implicit that setting standards in their reporting might have an impact on their conduction. Further, it has been previously reported that mentioning the QUORUM in the manuscript is associated with higher compliance to the QUORUM checklist [9].

In order to evaluate the impact of PRISMA endorsement on both the methodological quality and quality of reporting of systematic reviews and meta-analyses, published in scientific journals in the field of gastroenterology and hepatology (GH), we assessed:

1. if PRISMA endorsement led to an increase in quality of reporting and methodological quality of published systematic-reviews and meta-analyses (Objective 1).
2. If explicit PRISMA referencing in the manuscript is associated with increased quality of reporting and methodological quality (Objective 2).
3. If quality of reporting and methodological quality of published systematic-reviews and meta-analyses differ among GH journals endorsing PRISMA compared to those not in the same time period (Objective 3).
4. If manuscript length and number of studies included are associated with quality of reporting and methodological quality (Objective 4).

## Methods

### Assessing endorsement of PRISMA in GH journals

The list of medical journal in the GH category was acquired from Thomson Reuters Current Contents in Clinical Medicine using the proper field code (Gastroenterology & Hepatology). We first identified the GH journals publishing systematic reviews and meta-analyses in MEDLINE. Two assessors (NP and SB) independently examined the instructions for authors section of the website of the 74 GH journals retrieved, and extracted any text mentioning the “PRISMA” word. The search was performed between September 1^st^, 2012 and October 1^st^, 2012. For each journal we retrieved the impact factor (IF) value according to the Journal Citation Reports (JCR) of 2011. Editors of journals who endorsed PRISMA were then contacted in order to know the exact year of PRISMA endorsement in the instructions for authors.

### Selection process of the systematic reviews and meta-analyses published in GH journals

**Figure 1:**
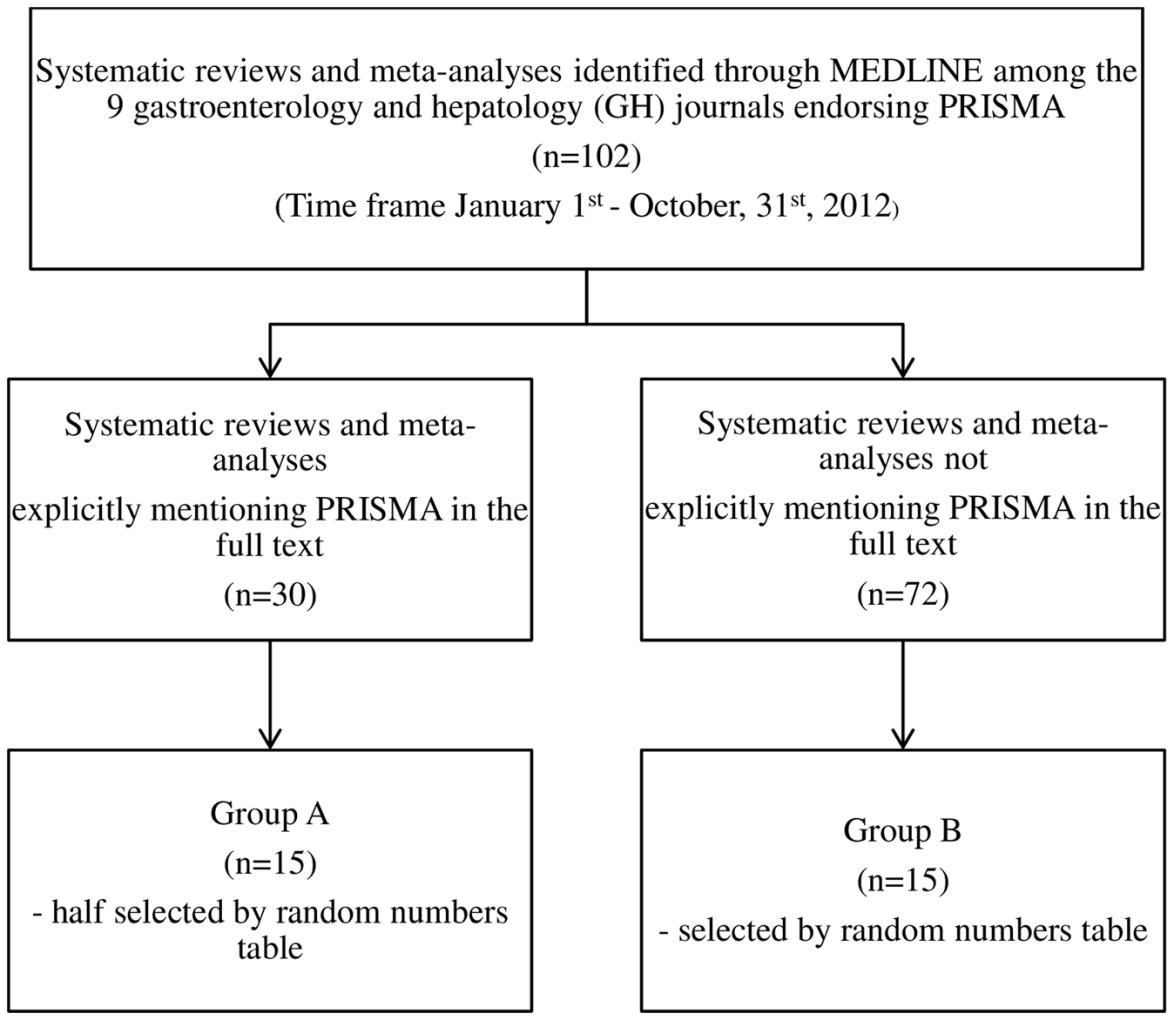
The search strategy and flow diagram for database search of systematic reviews and meta-analyses included in groups A and B, published in 9 GH journals endorsing PRISMA in the Instructions to Authors.

In order to identify the papers endorsing PRISMA, we used the following search terms in MEDLINE: ([journal name] AND [“systematic review”]) OR ([journal name] AND [“meta-analysis”]). The journal names were those explicitly endorsing PRISMA in the instructions for authors, and the time frame was from January 1^st^, to October, 31^st^, 2012. The Figure 1 depicts the flowchart of the search strategy and a diagram for the papers selection. One hundred and two relevant papers were identified, of which 30 explicitly mentioned PRISMA in the full text and 72 not. We than randomly selected half of the 30 papers to appraise (Group A; n = 15) whit explicit mention of PRISMA in the full text, and the same amount from the group of 72 papers (Group B; n = 15) with no explicit mention of PRISMA in the full text.

**Figure 2:**
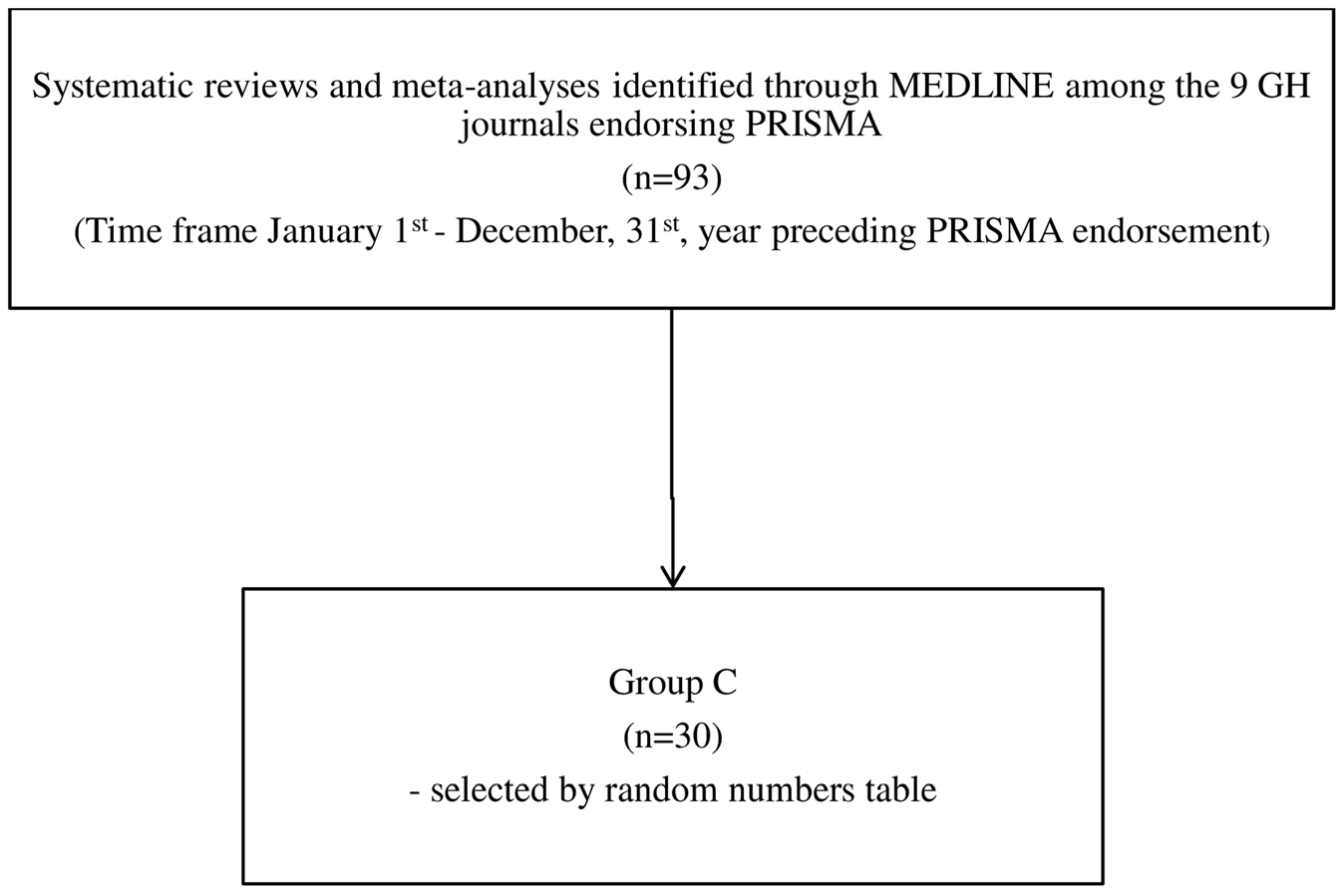
The search strategy and flow diagram of systematic reviews and meta-analyses included in Group C, published in 9 GH journal endorsing PRISMA in the year preceding PRISMA endorsement.

In order to identify the papers published in the GH journals endorsing PRISMA, in the year preceding the PRISMA endorsement, we used the following search terms in MEDLINE: ([journal name] AND [“systematic review”]) OR ([journal name] AND [“meta-analysis”]), with journal names as before, and the time frame referred to 1^st^ January–31^st^ December of the year preceding PRISMA endorsement for each journal. The Figure 2 depicts the flowchart of search strategy and a diagram for the papers selection. Ninety-three papers were identified and then 30 randomly selected (Group C).

**Figure 3:**
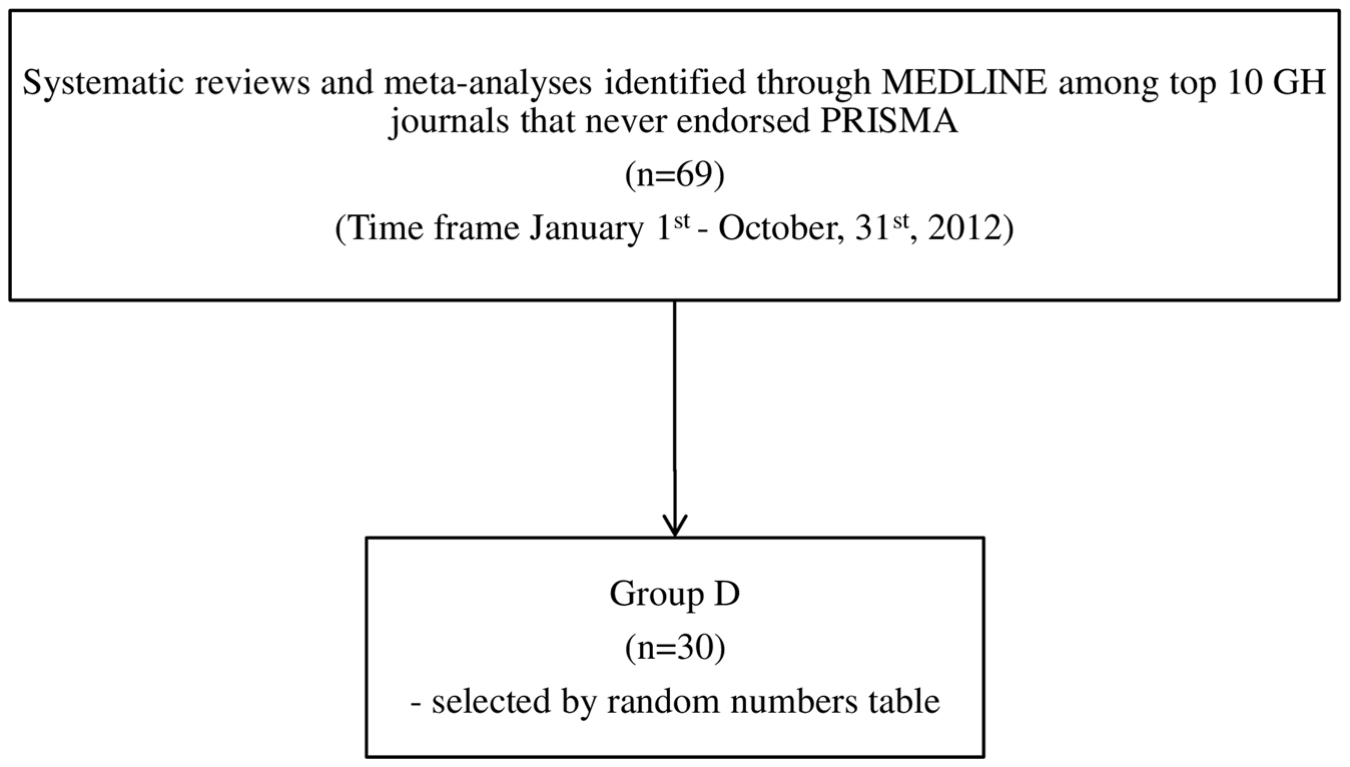
The search strategy and flow diagram for database search of systematic reviews and meta-analyses included in Group D, published in top 10 GH journals that never endorsed PRISMA.

In order to identify systematic reviews and meta-analyses published in the top 10 GH journals not endorsing PRISMA until 31^st^ December 2012, we used following search terms in MEDLINE: ([journal name] AND [“systematic review”]) OR ([journal name] AND [“meta-analysis]) with the time frame referred to 1^st^ January–31^st^ December 2012. The Figure 3 depicts the flowchart of search strategy and a diagram for the papers selection. Sixty-nine papers were identified and then 30 randomly selected (Group D). The IF, the number of pages and the numbers of primary studies included in 90 systematic reviews and meta-analyses selected was recorded.

We assessed the quality of reporting by evaluating the adherence of the papers to the PRISMA protocol, using the PRISMA checklist (Information S1) [8]. The methodological quality of the systematic reviews and meta-analyses was assessed by using a measurement tool for Assessment of Multiple Systematic Reviews (AMSTAR) scale (Information S2) [10]. Scoring the papers with PRISMA and AMSTAR checklists was performed by two researchers separately (NP and SB), and in case of disagreement an independent third researcher (GDB) was consulted.

Evaluating the adherence to the PRISMA checklist and compliance with AMSATAR scale was performed by assessing the adherence of each paper to the 27-items of the PRISMA checklist, and by evaluating the compliance with the AMSTAR items. We modified, however, the original 11-items of the AMSTAR scale, as we excluded two questions from the checklist. Questions no. 5 (“Was a list of studies (included and excluded) provided?”) and no. 11 (“Was the conflict of interest stated?”) were excluded as PRISMA guideline does not recommend authors to include the list of excluded studies in their manuscript, and some of the GH journals do not require authors to give declaration of the conflict of interest, respectively. We reported the results as % of adherence to PRISMA checklist and compliance with AMSTAR items, both overall as well as for each item particularly.

### Statistical analysis

Comparison of the data among groups was evaluated by the chi-square or Fisher test as appropriate. Medians and interquartile ranges were used to describe continuous variables and the nonparametric Wilcoxon rank-sum test was also used to compare the IF, number of pages and number of studies included among the groups. Statistical analysis was undertaken using Stata software (StataCorp. 2011. *Stata Statistical Software: Release 12*. College Station, TX: StataCorp LP).

## Results

### Assessing endorsement

Although all of 74 GH journals mentioned in the instruction for authors that they publish systematic reviews and/or meta-analyses, our MEDLINE search could not identify any systematic reviews or meta-analyses ever published in 4 journals, so they were excluded from the further selection process. Among the remaining, the PRISMA Statement was referred in the instructions for authors in 9 journals (12.9%; namely: Alimentary Pharmacology & Therapeutics, American Journal of Gastroenterology, BMC Gastroenterology, Colorectal Disease, Diseases Of The Colon & Rectum, Gut, Gut Pathogens, Hepatitis Monthly and HPB). Among them, 4 (44.4%) endorsed the statement in 2009, 4 (44.4%) in 2010, and 1 (11.1%) in 2012. The median IF of the nine GH journals endorsing PRISMA was 3.95 (range 1.60–10.10), compared with 2.93 (range 0.47–11.68) of the remaining 61 GH journals not endorsing PRISMA (p = 0.194, data not shown).

### Critical appraisal of systematic reviews and meta-analyses published in GH journals

In order to apprise if PRISMA endorsement led to an increase in quality of reporting and methodological quality (Objective 1), we compared the Group A versus Group C with the respect to adherence to PRISMA checklist and compliance with AMSTAR scale. Results are reported in Tables 1 and 2, showing significant differences among Groups A and C in both overall adherence to PRISMA checklist (Group A, 90.1% versus Group C, 83.1%; p = 0.003) as well as overall compliance with AMSTAR items (Group A, 85.0% versus Group C, 74.6%; p = 0.002). In particular, we observed a significantly higher rate of adherence to item no. 17 (“Study selection”) of PRISMA (Group A 100.0% versus Group C 63.3%; p = 0.008) and compliance with question no. 3 (“Was a comprehensive literature search performed?”) of AMSTAR (Group A, 93.3% versus Group C, 60.0%, p = 0.034).

**Table 1:** Evaluation of the adherence to PRISMA checklist of 90 systematic reviews and meta-analyses.

PRISMA item	Group A†	Group B‡	Group C○	Group D^i^
	(n = 15)	(n = 15)	(n = 30)	(n = 30)
1	100.0	100.0	90.0	73.3
2	100.0	100.0	100.0	56.7
3	100.0	100.0	100.0	100.0
4	100.0	100.0	86.7	100.0
5	6.7	13.3	3.3	0.0
6	100.0	100.0	90.0	90.0
7	100.0	100.0	93.3	93.3
8	53.3	40.0	50.0	56.7
9	86.7	93.3	73.3	79.3
10	86.7	80.0	76.7	79.3
11	93.3	93.3	76.7	79.3
12	100.0	100.0	86.4	100.0
13	100.0	100.0	100.0	100.0
14	83.3	100.0	100.0	95.0
15	100.0	100.0	100.0	100.0
16	100.0	100.0	84.6	100.0
17	100.0	93.3	63.3	83.3
18	93.3	93.3	83.3	93.3
19	81.8	81.8	61.9	87.5
20	93.3	100.0	95.0	100.0
21	100.0	100.0	100.0	100.0
22	87.5	100.0	88.9	100.0
23	100.0	100.0	100.0	100.0
24	100.0	100.0	96.7	100.0
25	93.3	100.0	93.3	96.7
26	100.0	100.0	100.0	100.0
27	na	100.0	100.0	100.0
Total (CI 95%)	90.1 (86.4–93.0)	91.1 (87.6–93.8)	83.1** (80.0–85.8)	85.3** (82.4–87.9)

CI: confidence interval na: non-applicable.

† = papers explicitly endorsing PRISMA in journals endorsing (or mandating) PRISMA.

‡ = papers not-explicitly endorsing PRISMA in journals endorsing (or mandating) PRISMA.

○ = papers published in journals endorsing PRISMA (the year preceding its endorsement).

i = papers from top 10 highest GH journals which never adopted PRISMA.

** p<0.01.

p-value calculated for each of these comparisons A vs B, A vs C, A+B vs D.

**Table 2:** A quality assessment of the 90 systematic reviews and meta-analyses using the AMSTAR checklist.

AMSTAR item	Group A†	Group B‡	Group C○	Group D^i^
	(n = 15)	(n = 15)	(n = 30)	(n = 30)
1	100.0	100.0	90.0	96.7
2	73.3	80.0	70.0	73.3
3	93.3	93.3	60.0	80.0
4	100.0	80.0	86.7	66.7
6	93.3	86.7	83.3	90.0
7	73.3	73.3	66.7	60.0
8	73.3	73.3	66.7	60.0
9	81.8	100.0	100.0	100.0
10	72.7	88.9	47.4	70.0
Total (CI 95%)	85.0 (77.6–90.7)	85.6 (78.2–91.2)	74.6* (68.7–79.9)	76.9* (71.2–82.0)

CI: confidence interval.

† = papers explicitly endorsing PRISMA in journals endorsing (or mandating) PRISMA.

‡ = papers not-explicitly endorsing PRISMA in journals endorsing (or mandating) PRISMA.

○ = papers published in journals endorsing PRISMA (the year preceding its endorsement).

i = papers from top 10 highest GH journals which never adopted PRISMA.

* p-value<0.05.

** p<0.01.

*** p<0.001.

p-value calculated for each of these comparisons A vs B, A vs C, A+B vs D.

Secondly, in order to evaluate if explicit PRISMA mentioning in the full text is associated with increased quality of reporting and methodological quality (Objective 2), we compared the Group A versus Group B. Results show that there was no difference in both the overall adherence to PRISMA and compliance with AMSTAR, as well as to any particular PRISMA or AMSTAR item (Tables 1 and 2). In that light, we joined Groups A and B together and compared it again with Group C, with difference in the overall adherence to PRISMA (p<0.001) and compliance to AMSTAR (p = 0.003) even increasing (data not shown).

Thirdly, in order to estimate if quality of reporting and methodological quality of published systematic reviews and meta-analyses differ among GH journals endorsing PRISMA and those not in the same time period (Objective 3), we compared Groups A and B jointly versus Group D. Significant difference was observed in the overall adherence to PRISMA (p = 0.003) and compliance with AMSTAR (p = 0.016) (Tables 1 and 2). In particular, PRISMA items no. 1 (“Title:) and no. 2 (“Structured abstract”) were addressed correctly in 100% of papers from Group A+B comparing to 73.3% (p = 0.005) and 56.7% (p<0.001), respectively, in Group D. Finally, AMSTAR question no. 4 (“Was the status of publication (i.e. grey literature) used as an inclusion criterion?”) was address correctly in 90% of papers from overall Group A+B compared with 66.7% from Group D (p = 0.028).

Overall, 27 (30.0%) out of 90 papers evaluated were systematic reviews and 63 (70.0%) were meta-analyses. The mean IF of the 90 papers was significantly higher in group C compared to group A (p = 0.023), as well as in the Group D compared to A+B (p<0.001) (Table 3). There was no significant difference among the papers included according to the length of manuscript, as well as the number of primary studies included (Table 3).

**Table 3:** Characteristics of 90 systematic reviews and meta-analyses evaluated.

	Group A†	Group B‡	Group C○	Group D^i^
	(n = 15)	(n = 15)	(n = 30)	(n = 30)
Impact factor	2.9 (2.9–3.8)	3.8 (2.9–7.3)	3.8 (2.9–7.3)*	5.4 (4.9–11.7)***
N_o_ pages	9 (8–12)	10 (9–11)	10 (8–13)	9 (8–10)
N_o_ studies	12 (9–17)	20 (11–33)	14 (7–31)	14 (10–23)

CI: confidence interval.

† = papers explicitly endorsing PRISMA in journals endorsing (or mandating) PRISMA.

‡ = papers not-explicitly endorsing PRISMA in journals endorsing (or mandating) PRISMA.

○ = papers published in journals endorsing PRISMA (the year preceding its endorsement).

i = papers from top 10 highest GH journals which never adopted PRISMA.

* p-value<0.05.

** p<0.01.

*** p<0.001.

p-value calculated for each of these comparisons A vs B, A vs C, A+B vs D.

Values are expressed as median and interquartile range.

## Discussion

Although a relatively small proportion of the scientific journals in field of GH endorsed PRISMA, we report that the quality of reporting and methodological quality of systematic reviews and meta-analyses in these journals have significantly increased after PRISMA endorsement. Our results show a significant increase in the overall adherence to PRISMA checklist and compliance to the AMSTAR scale items. The increased quality was observed for all the papers published on journals endorsing PRISMA, regardless of the fact that authors explicitly declared the adoption or not. Additionally, we showed that quality of reporting and methodological quality was higher in journals endorsing PRISMA compared to those not in the same time period.

It has been reported that in 2011, among 146 high-impact medical journals, more than half of general and internal medicine publications, and a quarter of other specialty journals referred to the PRISMA in the instructions for authors [9]. Our study showed that a much smaller portion of the GH journals has endorsed PRISMA in their instructions for authors until September 2012, independently from their IF values.

The impact of reporting guidelines on quality of published papers in medical journals is contradictory. It has been reported that endorsement of the Consolidated Standards for Reporting Trials (CONSORT) [11] have lead to an improved quality in the reporting of randomized clinical trials [12]–[14]. On the other hand, after endorsement of Strengthening the Reporting of Observational Studies in Epidemiology (STROBE) statement [15], the quality of reporting of published observational studies was still reported as unsatisfactory [16]. Results of one masked randomized trial suggested that additional paper reviews based on reporting guidelines can improve manuscript quality [17]. Moher et al [18] reported inconsistent quality of reporting among systematic reviews indexed in MEDLINE during November 2004, and suggested that endorsing and adhering to reporting-guidelines may improve the situation.

No study so far, however, tried to assess the impact of PRISMA endorsement both on quality of reporting as well as methodological quality of the systematic reviews and meta-analyses. Tao et al [9] examined to what extent high-impact medical journals endorsed PRISMA in 2011, but it was too early to evaluate authors' adherence to PRISMA checklist. Instead of, they addressed adherence of published manuscripts to the QUORUM statement, reporting insufficient adherence to several QUORUM items such as “Identifying title” and “Structured abstract” [9]. These are, among others, the exact fields in which we have found improvement after PRISMA endorsement. Further, although the PRISMA statement was primarily developed to improve the quality of reporting of systematic reviews and meta-analyses [7], we assumed that improving the quality of reporting can also influence their ideation and conduction. We report that PRISMA endorsement led not just to increased adherence to PRISMA checklist items, improving quality of reporting, but also improved compliance with AMSTAR scale items, supporting the idea that reporting guidelines can improve the methodological quality of the studies.

Tao et al [9] previously reported that mentioning of QUORUM in full text was associated with higher compliance with QUORUM checklist [9]. We did not find, instead of, any difference in both quality of reporting as well as methodological quality among systematic reviews and meta-analyses explicitly adopting PRISMA in the full text compared to those not. We also report that the overall quality of published systematic reviews and meta-analyses was higher in journals endorsing PRISMA compared to those not endorsing during the same time period, even though the mean IF was significantly lower in the first group.

Findings on association between manuscript length and quality of systematic reviews and meta-analyses have been contradictory so far [9], [19]. We did not find quality of papers to be associated both with manuscript length as well as with the number of studies included. We believe, however, that the length of systematic reviews and meta-analyses as well as the number of studies included primarily depends on the issue the reviews refer to, and are not expected to have a major impact on the quality of the paper.

We acknowledge that our study has some limitations. Our research was restricted on systematic reviews and meta-analyses published in GH journals, still we argue that our evidences might be also useful to other categories of journals who are not explicitly connected with this area of medicine. We were unable to retrieve information on the potential for a former adoption of QUORUM from the nine GH journals we identified endorsing PRISMA. If this was the case, it could be that QUORUM and not PRISMA affected the quality of published papers, though we tend to exclude it as we reported a significant difference between GH journals endorsing PRISMA and not in 2012, independently of any potential QUORUM adoption.

In conclusion, we report that endorsing PRISMA from scientific journals in field of gastroenterology and hepatology resulted in an improvement in the methodological quality and quality of reporting of published systematic reviews and meta-analyses. It is advisable that PRISMA is endorsed in the instruction for authors from a larger proportion of medical journals.

## Supporting Information

Information S1 **PRISMA checklist** [**10**] **.**
(TIF)

Information S2 **AMSTAR checklist** [**8**] **.**
(DOCX)
