# Supplementary figures and images for: Evaluation of the Endorsement of the Preferred Reporting Items for Systematic Reviews and Meta-Analysis (PRISMA) Statement on the Quality of Published Systematic Review and Meta-Analyses
Source: PLoS One. 2013 Dec 26;8(12):e83138. doi: 10.1371/journal.pone.0083138 (PMC3873291; doi:10.1371/journal.pone.0083138)

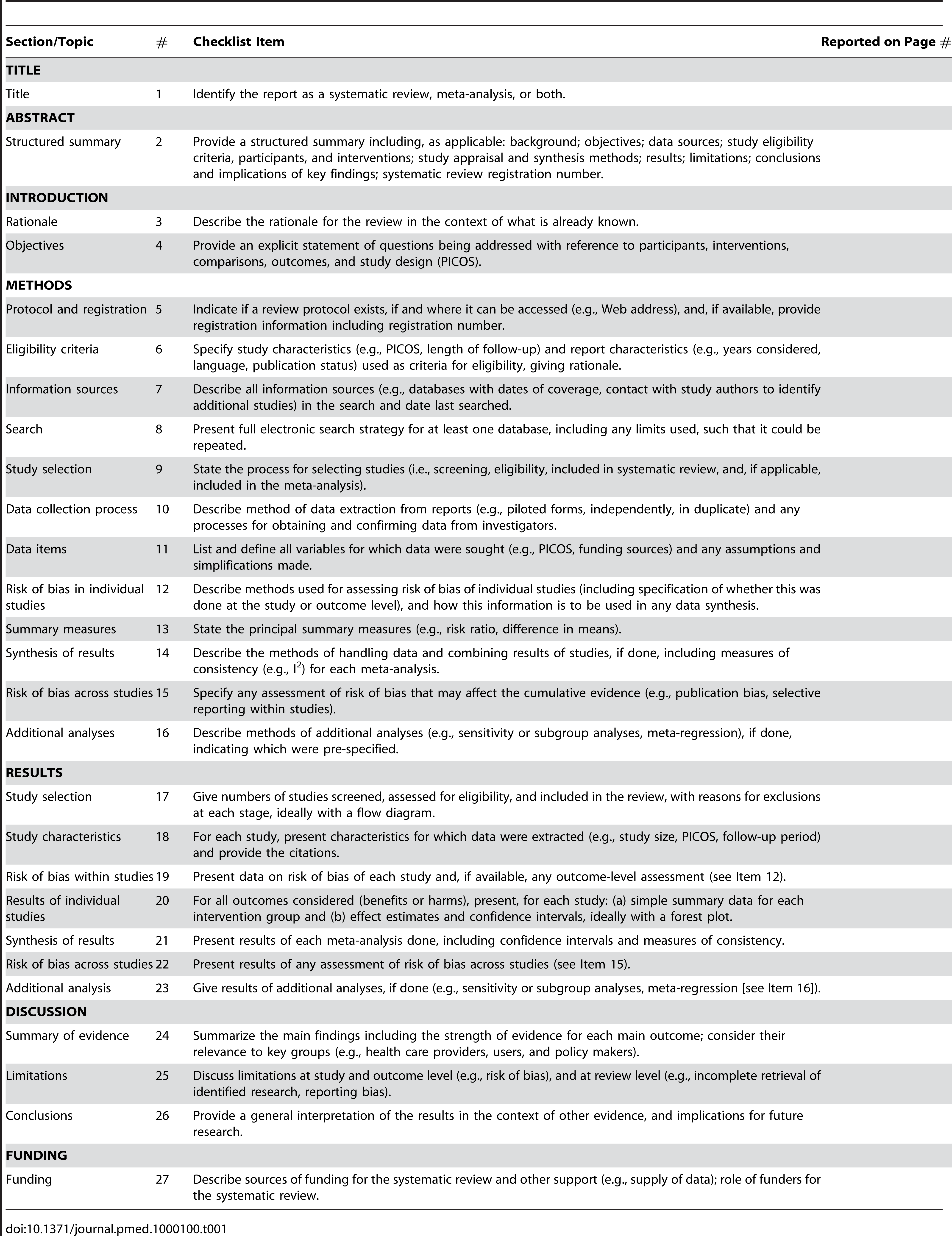

Supplement: Information S1 — PRISMA checklist [10] . (TIF) [file pone.0083138.s001.tif]
